# Do project management and network governance contribute to inter-organisational collaboration in primary care? A mixed methods study

**DOI:** 10.1186/s12913-018-3169-8

**Published:** 2018-06-07

**Authors:** Sanneke Schepman, Pim Valentijn, Marc Bruijnzeels, Marlies Maaijen, Dinny de Bakker, Ronald Batenburg, Antoinette de Bont

**Affiliations:** 1 NIVEL, Netherlands Institute for Health Services Research, PO Box 1568, 3500 BN Utrecht, the Netherlands; 2 Department of Health Services Research, School for Public Health and Primary Care, Faculty of Health, Medicine and Life Sciences, Maastricht University, PO Box 616, 6200MD Maastricht, the Netherlands; 3 Integrated Care Evaluation, Essenburgh Research & Consultancy, Hierden, The Netherlands; 4 Jan van Es Institute, Netherlands Expert Centre Integrated Primary Care, Randstad 2145-a, 1314 BG Almere, The Netherlands; 5 Institute of Health Policy & Management, Erasmus University Rotterdam, PO Box 1738, 3000 DR Rotterdam, the Netherlands; 6 Department of Sociology, Radboud University Nijmegen, P.O. Box 9104, 6500 HE Nijmegen, the Netherlands

**Keywords:** Inter-organisational, Collaboration, Governance, Project management, Patients, Professionals, Primary care

## Abstract

**Background:**

The need for organisational development in primary care has increased as it is accepted as a means of curbing rising costs and responding to demographic transitions. It is only within such inter-organisational networks that small-scale practices can offer treatment to complex patients and continuity of care. The aim of this paper is to explore, through the experience of professionals and patients, whether, and how, project management and network governance can improve the outcomes of projects which promote inter-organisational collaboration in primary care.

**Methods:**

This paper describes a study of projects aimed at improving inter-organisational collaboration in Dutch primary care. The projects' success in project management and network governance was monitored by interviewing project leaders and board members on the one hand, and improvement in the collaboration by surveying professionals and patients on the other. Both qualitative and quantitative methods were applied to assess the projects. These were analysed, finally, using multi-level models in order to account for the variation in the projects, professionals and patients.

**Results:**

Successful network governance was associated positively with the professionals’ satisfaction with the collaboration; but not with improvements in the quality of care as experienced by patients. Neither patients nor professionals perceived successful project management as associated with the outcomes of the collaboration projects.

**Conclusions:**

This study shows that network governance in particular makes a difference to the outcomes of inter-organisational collaboration in primary care. However, project management is not a predictor for successful inter-organisational collaboration in primary care.

**Electronic supplementary material:**

The online version of this article (10.1186/s12913-018-3169-8) contains supplementary material, which is available to authorized users.

## Background

The need to collaborate in primary care has been stressed for a long time. This need has two main drivers [1–5]. The first is the strong growth in the older population with multiple chronic physical and mental conditions [6], to whom different primary care professionals provide care. The second driver is rising costs. Collaboration can contribute to keeping care sustainable and affordable by making it easier to shift patients from both hospital and acute care to ambulatory and preventive care [7].

In many high-income countries primary care is still provided in small mono-disciplinary practices [5], however, a trend has been observed towards increasing the scale of such facilities. Key examples are: the establishment of larger mono-disciplinary group practices; multidisciplinary health centres; and care groups for disease management [8, 9]. A further, alternative but parallel development, has occurred next to the growth of these larger primary care organisations. This is the collaboration between small mono-disciplinary practices.

This development of inter-organisational collaboration in primary care does not seem to be an autonomous or natural process. It is typically initiated by bundled payments to stimulate collaboration between organisations and by innovation projects [10, 11] that are focused on improving the position of professionals and patients. The innovation projects often have specific goals, such as improving the care of patients with diabetes, and more general goals, such as strengthening collaboration between the participating organisations and in doing so reducing costs.

Thus, inter-organisational collaboration may not be the goal, but the final outcomes are key to judging the success of the projects. Patients are dependent on the quality of care and this quality of care is, for the most important part, provided by the health care professionals. The judgement of professionals and patients is, therefore, important in gauging the success of the project.

To achieve their goals, *project management* is used as a method or ‘tool’ in such innovation projects [12]. Project management is the application of processes, methods, knowledge, skills, and experience, by project leaders and members, to achieve the project’s objectives [12]. In addition to project management, network governance is generally considered as an important method to enhance inter-organisational collaboration, especially in complex settings [13]. A certain level of governance is necessary in order for more organisations to collaborate. *Network governance* comprises steering and management strategies aimed at managing the complex problems in an interdependent setting with many different actors [13]. It is used to align the project’s aims and activities. Literature shows that successful network governance is key to sustaining collaboration between organisations over time [14, 15]. It contributes to the strength of the network, critical to the success of the project [15]. Network governance depends on four factors: the number of participants; the trust among participants; having a consensus among board members about their goal; and the network competencies of the board members [15].These four variables of network governance, and, therefore, the form of network governance used, should fit the type of project. Choosing the right form determines the success of network governance [15].

We explore, in this paper, the validity of these statements by evaluating the success of projects in Dutch primary care, which were the first to initiate inter-organisational collaboration.

The central research question of this paper is: *Do project management and network governance relate to the improvement of outcomes in inter-organisational collaboration in primary care, as experienced by professionals and patients?*

## Methods

### The collaboration projects in primary care

We analysed projects in the Netherlands for this paper that were part of a national ‘Primary Focus programme’ (PF) [16]. This PF programme, coordinated by ZonMw (the Netherlands Organisation for Health Research and Development), was initiated in 2009 by the Dutch Ministry of Health, Welfare and Sport. It aimed to enhance and stimulate organisational development and innovation of local primary care services. ZonMw selected projects in primary health care, developed locally, for funding which met the following criteria:

1. The goal of the project was to build organisational capacity by improving multidisciplinary collaboration;
2. The organisational capacity built up by the project was aimed at improving the quality, accessibility, efficiency, and transparency, of services in primary care;
3. The project focused on community care in a neighbourhood, a village, a city or a region;
4. The initiative’s project team was multidisciplinary;
5. The project was aimed at a sustainable organisational structure after the project had been completed;
6. The project contributed to new knowledge about organisational structures and developments in local health care.

Most of the projects were initiated by one or more managing directors or managers of the organisations participating. Representatives of the organisations participated in a steering committee. All projects started between 2010 and 2012. The projects differ by the following: goal, such as development or implementation; size, such as a collaboration between two disciplines or a collaboration between 30 organisations; duration, such as between one to 4 years; subject, such as care for specific chronic diseases, primary mental health care, care for the elderly or integration between welfare and primary health care; and expected outcomes, such as improved service, or improved health status [17].

### The monitor

**Fig. 1:**
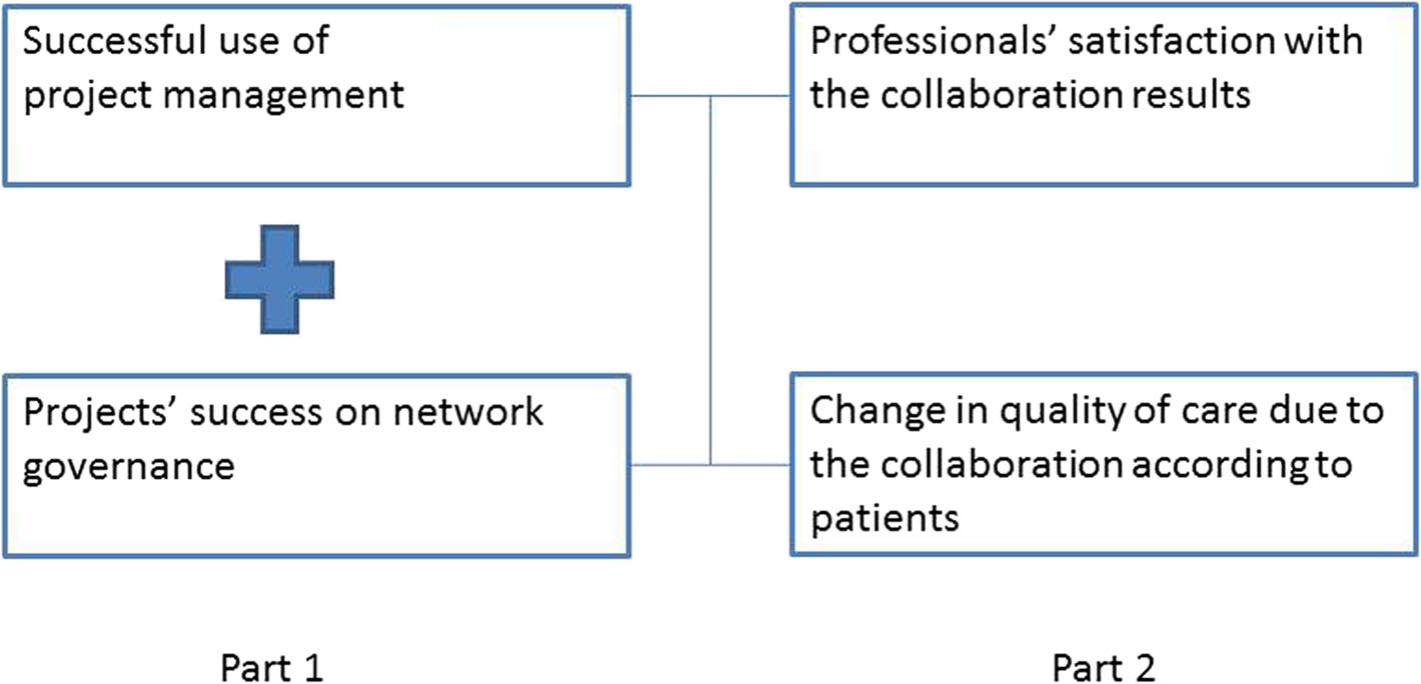
Schematic representation of the analysis

A research project, which was also financed by ZonMw, monitored the structure, process and outcomes of the projects of the PF Programme. This meant the research question could be answered about whether the project management and network governance are indeed determinants of the project’s success in primary care. A schematic representation of the analysis is shown in Fig. 1. Our expectation is that ‘part 1’, the left-hand side of the figure, will increase the outcomes of ‘part 2’, on the right-hand side of the figure. These outcomes are the satisfaction of health care professionals with the project results and the quality of care according to patients.

### Professionals’ satisfaction with the collaboration outcomes

Surveys were conducted which measured satisfaction with the collaboration of the projects from the perspective of the *professionals* involved. The surveys were based on earlier research [18, 19]. To get a high response the questionnaire contained only a small amount of questions and therefore a strict selection of questions from the literature was done. The surveys were sent to health care professionals at the start and the end of the project’s period of funding, either by email or mail depending on their preferences. For this analysis, we only used the surveys sent at the end of the projects in order to focus on the measurement of the outcomes. The questionnaire contained 20 questions, but for this paper the following question was used: “How satisfied are you with the results of the collaboration?” The answers could be any of: “very unsatisfied, unsatisfied, neutral, satisfied, very satisfied”.

### Change in the quality of care due to the collaboration, according to patients

A survey was also conducted among *patients* who were connected to the projects. At the start and the end of the project’s period of funding, surveys were distributed randomly on paper by health care professionals to a sample of a maximum of 100 patients involved per project. For the same reason as stated before, we only used the survey data collected at the end of the project. The patient survey contained 13 questions. Some questions in the survey were based on questions in the experiences CQI questionnaire used in primary care [20]. The quality of care according to patients was measured by the following key question posed at the end of the project: “Has the quality of care changed due to the project?” The answers could be: “it has improved, it remained the same, it has become worse”.

### The projects’ successes in project management

The success of the project management dimension was rated by interviewers who were carrying out the monitoring. They interviewed, per project, the *project leader and the two project managers* at three stages during the project - at the beginning, the mid-term and at the end. The interviews were semi-structured and focused on the realisation of the project’s goals. Based upon their notes, the interviewers filled in a questionnaire with closed-ended questions. The interviewers were trained to judge the project management in an independent and comparative manner. The interviewers assessed the project management at the end of the project simply by answering the following question: “How successful was the project management based on your interviews with project leaders and project team members?” [21] On a 5-point scale they could be: “very successful, successful, neutral, unsuccessful, not successful at all”.

### The projects’ successes in network governance

Questionnaires were conducted among directors and/or representatives of the organisations who participated in the projects’ steering committee in order to measure the success of network governance. The questionnaires were aimed at supporting the meetings of *board members* in their efforts to provide the participants with feedback [17]. Participants were asked to complete a short questionnaire before meetings - at the beginning of the project, half way through it and the end. This would allow them to reflect on both the collaboration process and on their own and shared interests. How board members perceived its success was investigated at the end of the project using as a 10-point grading scale: 1 = the lowest score of the project success, 10 = the highest score. This resulted in a mean score per project for network governance as perceived by board members.

#### Statistical analysis

To account for the hierarchical structure of the data, a multilevel analysis was used. The data were structured on two levels, the professionals and the patients’ level. These were nested within the second level: the project. Two different models were used. In the first model the outcomes for professionals were tested and in the second model the outcomes for patients. Data were analysed using Stata 13.1 and MLwiN version 2.30. A significance level of 0.05 was used for all statistical tests.

## Results

### The collaboration projects in primary care

Data were collected from 69 collaboration projects in the PF programme in the Netherlands. More information about the focus and organisation of the projects can be found in Additional file 1. These projects monitored patients, professionals, project leaders and steering committees.

### The monitor

Not every measurement in all projects could be included in the monitor. In 12 projects, the survey among professionals could not be sent at the end as they had either stopped beforehand or the project team had not cooperated with this part of the research. The response rate of all the surveys sent to the professionals was 46%. In total 714 questionnaires were filled in by professionals who were involved in 47 projects.

The patients’ surveys could not be carried out in 21 projects, largely because they were not able to understand or complete the questionnaire. Among the remaining projects, the response rate to the survey among patients of was 30%. This amounted to 788 completed questionnaires from patients in 31 projects.

All projects and project leaders participated in the interview, resulting in nine interviews per project. In this way, a project management success score for every project was achieved.

On average five board members per project filled in the questionnaires. By the end of the project 78% of the board members had responded (229 out of 294 questionnaires).

Descriptive statistics show that among the 69 projects there was considerable difference between the projects’ success on project management and network governance, on the one hand, and the outcomes of the project as perceived by patients and professionals on the other (Table 1).

**Table 1:** Success of the project: the perception of professionals, patients, project managers and board members

	mean	sd	N	Range
Success, according to health care professionals, in their satisfaction with the outcomes regarding collaboration at the end of the project	3.6	.69	327	1-5
Success, according to patients, in the change in quality of care between the start and end of the project	2.2	.47	226	1-3
Success, according to interviewers, in the project management at the end of the project	3.6	.91	63	1-5
Success, according to board members, in the network governance at the end of the project	7.1	1.63	55	1-10

Measured at the end of the project, 6% of the professionals indicated that they were not satisfied with the results of the collaboration, 34% were neutral and 60% were satisfied (mean score 3.6). Patients appeared to be more neutral as 74% of them indicated that the quality of care did not change during the project, 4% indicated a decrease, and 23% an increase (mean score 2.2). Almost 20% of the interviewers rated the project management as neutral, 67% perceived it as successful, and 15% thought it was unsuccessful, or very unsuccessful (mean score 3.6). For network governance on a scale from one to ten, 16% of the board members rated the collaboration at the end of the project lower than 6, almost 15% rated it 6, and almost 70%, 7 or higher (mean score 7.1).

### Professionals’ satisfaction with the collaboration outcomes

Table 2 shows the results of a multilevel analysis. Satisfaction with the collaboration as experienced by professionals is the dependent variable. The assessed project management and network governance of the project are the independent variables. The age and gender of the professionals are included as control variables.

**Table 2:** Multilevel analysis: satisfaction with the results of the collaboration according to professionals at the end of project

	Professionals’ satisfaction with results T2	
Response	B	S.E.
Fixed Part		
Constant	2.941	(0.364)
Female^1^	−0.09	(0.094)
Age	−0.002	(0.004)
Success of project management according to interviewers	0.057	(0.065)
Success of network governance according to project board members	0.087*	(0.035)
Random Part		
Level: project		
cons/cons	0.006	(0.015)
Level: professional		
cons/cons	0.454*	(0.042)
ICC^2^	0.013	
-2*loglikelihood:	540.142	
N project	41	
N professional	262	

^1^ref cat: Male

^2^ICC = Intra Class Correlation

*p < .05

The projects that were assessed as succesful, with regard to network governance score, significantly higher (B = .087, *p* < .05) on the professionals’ satisfaction with the collaboration. Success of project management, however, is not related to the project outcomes according to professionals. The random part of the multilevel model shows that outcomes were not due to nesting within projects, but were explained by differences between professionals (ICC = 0.013). As control variables, neither the gender, nor age, of the professionals show significant relationships with the dependent variable.

### Change in the quality of care due to the collaboration, according to patients

Table 3 shows the results of a second multilevel analysis of the quality of care, as experienced by patients.

**Table 3:** Multilevel analysis: change in the quality of care according to patients at the end of the project

	Change in the quality of care by patients at T2	
Response	B	S.E.
Fixed Part		
Constant	2.499	(0.393)
Female^1^	−0.018	(0.078)
Age	−0.006*	(0.002)
Success of project management according to interviewers	−0.012	(0.064)
Success of network governance according to project board members	0.015	(0.039)
Random Part		
Level: project		
cons/cons	0.015	(0.012)
Level: patient		
cons/cons	0.205*	(0.021)
ICC^2^	0.068	
-2*loglikelihood:	261.758	
N project	19	
N patient	201	

^1^ref cat: Male

^2^ICC = Intra Class Correlation

*p < .05

Table 3 shows that, according to patients, neither the assessed success of project management, nor the success of network governance, is significantly related to the change in the quality of care due to the project.

The control variable, age, appears to have a statistically significant negative relationship to the dependent variable. This implies that older patients perceived, more often, a decrease in the quality of care of the project. The greatest degree of unexplained variance is located on the level of the patient (ICC = 0.068), meaning that the differences found are due to differences among patients and not to the projects.

## Discussion

The success of inter-organisational projects in primary care was measured in this study by four elements. These were: (1) the satisfaction with the collaboration of the project according to professionals; (2) the improvement in the quality of care during the project according to patients; (3) the project managements’ success based on interviews with three project managers, and; (4) the degree of success network governance achieved according to board members. Projects with successful network governance gained higher scores for the satisfaction of the professionals with the inter-organisational collaboration; but projects with successful project management did not. Neither the success of project management, nor that of network governance appears to be positively, and significantly, related to the quality of care as experienced by patients.

The outcomes with regard to network governance align with a recent review of national improvements in the quality of health care in the United Kingdom [14] and the Netherlands [21, 22]. Both reviews/studies showed network governance as a success factor for implementing projects geared towards innovation in the quality of health care. Our outcomes with regard to the lack of importance of project management may be explained by the complexity of collaboration between several organisations each with their own infrastructures [15]. In such complex projects, network governance is possibly more important than project management.

Professionals and patients might not always have been aware of the projects, however, as most of the projects were initiated by managing directors or managers themselves who often made the project plan and took decisions as well. Hence, the success of project management according to the interviewers, as well as the success of network governance according to board members, appears to be out of step with the experiences of the professionals and patients [14].

We have two explanations as to why patients perceived, to a lesser degree, quality improvement as an outcome of the project compared to the professionals.

Firstly, while professionals could be directly influenced in their day to day work by the projects, patients are only affected indirectly. Consequently, health care professionals are actually an intermediate factor for the patients’ perception of quality. Secondly, it appeared that the age of patients was strongly related to the quality of care as perceived by patients. Older patients judged that the quality of care decreased more often due to inter-organisational collaboration. It is worthy of note that this result is not in line with earlier research where in most cases the satisfaction of older patients does not significantly differ from younger patients, although their preferences are less strong [23]. It might be that improvements in care through inter-organisational collaboration is more difficult to establish for this complex group of patients, who may frequently suffer from multiple morbidities. While the growth of multi-morbidity is actually one of the drivers behind stimulating inter-organisational collaboration, the result among older patients certainly calls attention for further research.

### Limitations

This study adds to the literature since it is based on the analysis of a substantial number of projects. Nevertheless, the research design also has a number of limitations. The projects that were monitored were very diverse in type, size, subject and intended outcomes. While this is an advantage for mapping and explaining factors that go beyond one, or a few, interventions, there is also a risk that the variation in types of collaboration is actually ‘too large’. Very large variation makes it difficult to account for a broader range of relevant confounders. Still, our analysis focused on common denominators - testing if there is a relationship between, project management, network governance, and project success for professionals and patients. By limiting the explanatory analysis to these variables, and including a limited number of control variables, the potential problems in variation were overcome. Also, by using a multilevel analysis, variation was explicitly taken into account. It is important to note that the variation in the project outcomes was not explained by the variation, or differences, between projects.

Another possible limitation is that the projects members were provided with feedback by the researchers during the monitoring. This could have influenced the direction and outcomes of the projects that are being studied [21]. While this interference is uncommon in study designs such as randomised controlled trials, it is quite common in evaluation studies of complex health interventions [11, 24]. Monitoring is mostly a part of the interventions designed to improve the project process by drawing on information which is gathered systematically. For these kinds of interventions, which seek to improve outcomes despite high levels of uncertainty, it is necessary to understand the complex interplay between context, structure, process and outcomes [25–28].

## Conclusions

Network governance is an important factor to take into account when studying inter-organisational collaboration. Project management as a tool seems to make no difference to the outcome of the project due, probably, to the complex structure of inter-organisational collaboration. Therefore, structural interventions are needed to embed inter-organisational collaboration in practice.

## Additional file

Additional file 1: Contains data that describe the projects and their diversity in participating organisations and patient groups. (DOCX 23 kb)

## Abbreviations

BM: Board members
ICC: Intra class correlation
NG: Network governance
PM: Project management
