# Supplementary material for: Do project management and network governance contribute to inter-organisational collaboration in primary care? A mixed methods study
Source: BMC Health Serv Res. 2018 Jun 7;18:427. doi: 10.1186/s12913-018-3169-8 (PMC5992666; doi:10.1186/s12913-018-3169-8)
Supplement: Supplementary file 1 — Contains data that describe the projects and their diversity in participating organisations and patient groups. (DOCX 23 kb) [file 12913_2018_3169_MOESM1_ESM.docx]

**Appendix 1 Description of the projects**

Figure 1 shows that general practices took the most frequent part in the projects’ steering committee, followed by home care and welfare organisations. Figure 2 shows that most of the 69 projects were aimed at patients in general, frail elderly or chronically ill.

**Figure 1 Participating organisations in the steering committee (Percentage of 69 projects)**

**Figure 2 Target patient groups of the project (percentage of 69 projects).**
